# Letter to Editor regarding “Effects of nutritional education intervention on frailty status in lung cancer patients”

**DOI:** 10.1016/j.jnha.2025.100707

**Published:** 2025-10-10

**Authors:** Parth Aphale, Shashank Dokania, Himanshu Shekhar

**Affiliations:** a Professor & Department Head, Dr. D.Y. Patil Vidyapeeth (Deemed to be University), Pimpri, Pune, Maharashtra, India; b Dr. D.Y. Patil Vidyapeeth (Deemed to be University), Pimpri, Pune, Maharashtra, India

Dear Editor,

We commend Li et al. for their timely and significant randomized controlled trial, "Effects of nutritional education intervention on frailty status in lung cancer patients: A randomized controlled trial," which addresses a critical need in geriatric oncology [1]. The study's focus on a vulnerable population and its demonstration of a positive impact on frailty and nutritional status through a non-pharmacological approach is highly valuable. However, as scholars and clinicians, we believe several methodological and interpretational points warrant further discussion and clarification.

A primary concern is the potential for observer and performance bias due to the lack of blinding for the investigators and data collectors. The study's design states that participants were blinded, but the individuals administering the intervention and assessing the outcomes were not. For outcomes such as self-reported physical activity (assessed by the IPAQ-SF), exhaustion, and quality of life (SF-12), the investigators' awareness of group assignment could inadvertently influence data collection or participant responses. Given the reliance on self-reported measures, this lack of blinding represents a substantial methodological limitation that could overestimate the observed effects [2]. We encourage the authors to comment on the measures they took to mitigate this potential bias.

Furthermore, we seek clarification on the nuanced interpretation of the results regarding frailty. The authors report a significant improvement in the mean frailty score for the intervention group, yet they also state that there was no significant difference in the *prevalence* of frailty between the groups after 12 weeks. This raises a crucial question about the clinical meaningfulness of the observed change. While a lower frailty score is a positive indicator, the failure to transition a significant number of patients from a frail to a pre-frail or robust state suggests the intervention may be more effective at mitigating decline than at reversing frailty within this timeframe. This distinction is vital for clinical application, and we would welcome the authors' perspective on how a reduction in score, without a change in prevalence, should be translated into practice [3].

Finally, while the multi-component nature of the intervention including personalized counseling, group sessions, and motivational calls appears to be effective, it presents a challenge for future implementation and research. The combined effect of these elements, while impactful, makes it difficult to ascertain which specific components drove the most significant improvements. This "black box" approach limits the ability to design more efficient or targeted interventions in the future [4]. A future study with a factorial design could help dissect the contributions of each component.

In conclusion, this research is a significant step forward in frailty management for lung cancer patients. Addressing these points will further strengthen the findings and contribute to the ongoing scholarly discourse on rigorous trial design and the clinical application of research in this field.

## CRediT authorship contribution statement

HS- Analysis, Interpretation, Writing the manuscript. PA- Data acquisition, Writing the manuscript. SD- Conception and Design, Final review, Conceptualization.

## Funding

None.

## Declaration of competing interest

The authors declare that they have no known competing financial interests or personal relationships that could have appeared to influence the work reported in this paper.
